# Synthesis, Characterization, and Antiproliferative Activity of Novel Chiral [QuinoxP*AuCl_2_]^+^ Complexes

**DOI:** 10.3390/molecules25235735

**Published:** 2020-12-04

**Authors:** Adedamola S. Arojojoye, R. Tyler Mertens, Samuel Ofori, Sean R. Parkin, Samuel G. Awuah

**Affiliations:** 1 Department of Chemistry, University of Kentucky, Lexington, KY 40506, USA; adedamolaarojojoye@uky.edu (A.S.A.); randall.mertens@uky.edu (R.T.M.); samuel.ofori@uky.edu (S.O.); s.parkin@uky.edu (S.R.P.); 2 Department of Pharmaceutical Sciences, College of Pharmacy, University of Kentucky, Lexington, KY 40536, USA

**Keywords:** gold, cisplatin, auranofin, antiproliferative, anticancer activity, ligands, QuinoxP*

## Abstract

Herein is reported the synthesis of two Au(III) complexes bearing the (*R*,*R*)-(–)-2,3-Bis(*tert*-butylmethylphosphino)quinoxaline (*R*,*R*-QuinoxP*) or (*S*,*S*)-(+)-2,3-Bis(*tert*-butylmethylphosphino)quinoxaline (*S,S*-QuinoxP*) ligands. By reacting two stoichiometric equivalents of HAuCl_4_.3H_2_O to one equivalent of the corresponding QuinoxP* ligand, (*R*,*R*)-(–)-2,3-Bis(*tert*-butylmethylphosphino)quinoxalinedichlorogold(III) tetrachloroaurates(III) (**1**) and (*S*,*S*)-(+)-2,3-Bis(*tert*-butylmethylphosphino)quinoxalinedichlorogold(III) tetrachloroaurates(III) (**2**) were formed, respectively, in moderate yields. The structure of (*S*,*S*)-(+)-2,3-Bis(*tert*-butylmethylphosphino)quinoxalinedichlorogold(III) tetrachloroaurates(III) (**2**) was further confirmed by X-ray crystallography. The antiproliferative activities of the two compounds were evaluated in a panel of cell lines and exhibited promising results comparable to auranofin and cisplatin with IC_50_ values between 1.08 and 4.83 µM. It is noteworthy that in comparison to other platinum and ruthenium enantiomeric complexes, the two enantiomers (**1** and **2**) do not exhibit different cytotoxic effects. The compounds exhibited stability in biologically relevant media over 48 h as well as inert reactivity to excess glutathione at 37 °C. These results demonstrate that the Au(III) atom, stabilized by the QuinoxP* ligand, can provide exciting compounds for novel anticancer drugs. These complexes provide a new scaffold to further develop a robust and diverse library of chiral phosphorus Au(III) complexes.

## 1. Introduction

The therapeutic application of metals is an ancient practice, dating back to the discovery of metals by man. Synthesis and application of metal complexes took a dramatic shift since the work of Rosenberg, Van Camp and Krigas on cisplatin were published [1]. This discovery is commonly referred to as the bedrock of metals in medicine [2]. Over the years, a few metal-based drugs such as Pepto-Bismol^®^ (Bismuth subsalicylate), Camcolit (Li_2_CO_3_), Flamazine (Silver sulfadiazene) and Auranofin (Gold) have been approved for the treatment of ulcer, manic depression, bacterial infection and arthritis [3,4,5], respectively. Recent studies on auranofin has been geared toward repurposing the drug as an anticancer agent [6,7].

Cancer is a leading cause of death globally, and the number of new cases of cancer in the United States is projected to be about 2 million in the year 2020 [8,9]. The number of metal-based complexes currently in use for the treatment of cancer is limited to a few platinum complexes (Platinol ^®^ and Paraplatin^®^) and Arsenic dioxide (Trisenox^®^) approved by the Food and Drug Admistration (FDA) [3,10,11], hence the need to develop new metallodrugs for use as anticancer agents. Our lab has been interested in gold metal complexes and their mechanism of action as novel possible anticancer agents [12,13]. We have synthesized and studied a library of gold(I)/(III) compounds with anticancer and antimicrobial activities with a view of understanding their mechanism of action and selectivity for tumor cells [14]. Using 1,2-Bis-(diphenylphosphino)benzene and 1,2-Bis[(2*S*,5*S*)-2,5-dimethylphospholano]-benzene ligands, gold(I) complexes with linear and square planar geometries have been synthesized with excellent antifungal activities in a panel of 21 *Candida* strains and 4 filamentous strains of *Aspergillus* spp and *Fusarium* spp [14,15]. Additionally, using (1*R*,2*R*)-(+)-1,2-diaminocyclohexane (DACH) ligands, six cyclometalated gold complexes were synthesized with IC_50_ in the micromolar range. These compounds were stable under physiological conditions with minimal effect from glutathione and sodium ascorbate [12].

**Figure 1:**
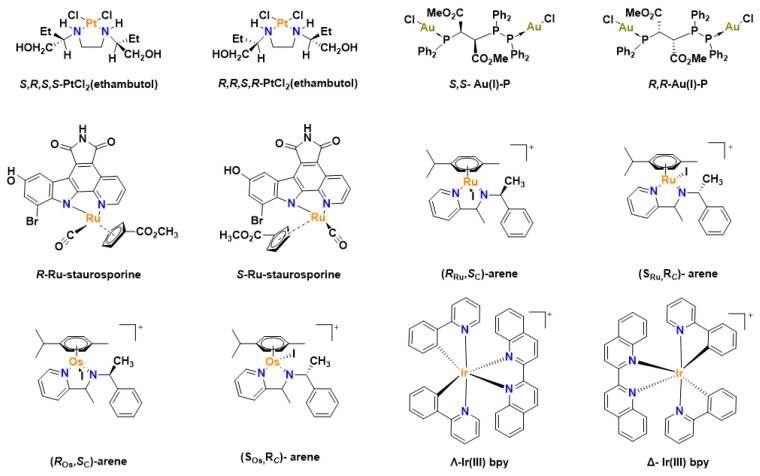
Chiral organometallic complexes with anticancer properties.

Chirality is a major point of concern when discussing the biological application of molecules. For example, all amino acids in the human body are chiral except for glycine which is achiral and all chiral amino acids exist as L-isomers in eukaryotes [16]. For effective actions, chiral drugs bind to specific chiral biomolecules in the body and a racemate or the enantiomer of the compound may have no effect or produce a deleterious effect in cells [17]. Ethambutol is an optically active compound used for the treatment of tuberculosis with only the *S*,*S* configuration active as an antibiotic, while the *R*,*R* compound is inactive [18,19]. Chiral enantiomeric platinum compounds have been reported to display different biological activities in different configurations [20,21]. Coordination of optically pure ethambutol ligand to platinum leads to two different isomers—*S*,*R*,*S*,*S*-PtCl_2_(ethambutol) and *R*,*R*,*S*,*S*-PtCl_2_(ethambutol) (Figure 1), with the nitrogen atoms becoming stable with either an *S* or *R* configuration and the ensuing platinum complexes possessing different biological activities [22]. This shows that the chirality of metal complexes is intrinsic to their biological characteristics [23]. Additionally, the configuration of the FDA approved anticancer drug oxaliplatin is (1*R*,2*R*)-cyclohexane-1,2-diamine](ethanedioato-*O*,*O’*) platinum(II), which exhibited lower mutagenicity but have a higher anticancer activity against colon cancer while the *S*,*S* isomer exhibits higher mutagenic activity but lower antitumor activity [17,24].

Staurosporine ligands coordinated with ruthenium have been studied as protein kinase inhibitors [25]. The *R*-enantiomer was a more potent inhibitor exhibiting a more than 250-fold increase in comparison to the S-enantiomer [25]. Ru(II) arene chiral complexes have also been studied, with the *R*,*S* or *S*,*R* isomers showing higher cytotoxicity than the *R*,*R* or *S*,*S* compounds in human ovarian cancer cell line A2780 [26]. Other chiral organometallic complexes that have been studied for their anticancer properties include osmium [27,28], iridium [29,30], rhodium [31], metallocene [32,33,34,35,36,37] and gold complexes [38]. Chiral N-Heterocyclic carbene (NHC) gold complexes have been reported to exhibit antitumor activity [38], while enantiomeric complexes of gold(I) with a phosphorus stereogenic center has shown great cytotoxicity in both suspended and adherent cells but with marked differences in their toxicity to healthy cells, with the *R*,*R* complex being more toxic than the *S*,*S* complex in mammalian cells [39,40].

Herein, we report the design and synthesis of two chiral Au(III) complexes bearing the chiral QuinoxP* ligand. Preliminary investigation into the biological activities of these two enantiomers shows no great difference in contrast to other enantiomeric platinum, ruthenium and osmium complexes.

## 2. Results and Discussion

### 2.1. Synthesis and Characterization

**Scheme 1:**
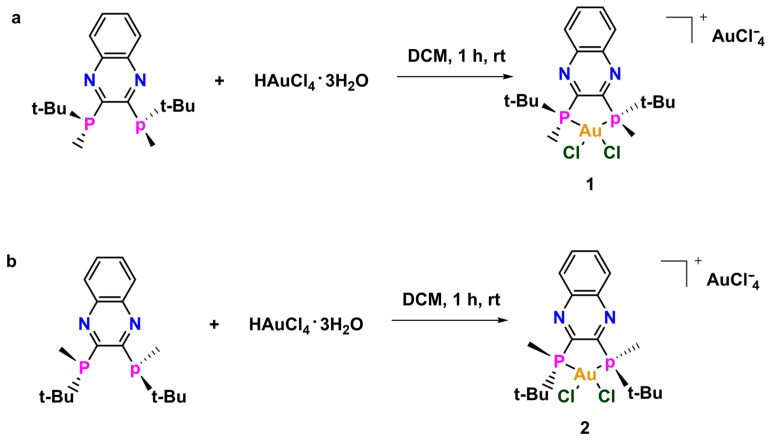
Synthesis of (**a**) **1** and (**b**) **2**.

Compounds **1** and **2** were synthesized by stirring the corresponding QuinoxP* with HAuCl_4_·3H_2_O in dichloromethane. The initial brick red color gradually changed to light yellow over the course of the reaction. The compounds were both precipitated from a concentrated DCM solution with excess Et_2_O to afford the yellow solids in good yields (Scheme 1). The compounds were characterized by ^1^H NMR, ^13^C NMR, ^31^P NMR, high-resolution mass spectrometry (HRMS) and the purity was ascertained by elemental analysis (EA). The structure of **2** was further confirmed by X-ray crystallography, indicating that a monocation complex was formed. At the onset, we hypothesized that the biological activities of the two chiral compounds will be different because building blocks (e.g., amino acids) in living systems themselves are chiral and each enantiomer of a chiral complex can behave in different ways due to potentially variant complex–protein interactions. Given the scarcity of chiral bisphosphine gold complexes used in biology and to build on our initial studies [14,15] in this area, we envisaged that changing the stoichiometric ratio of the reactant could lead to structures with unique geometries. The unique geometry discussed in this research provides more opportunity for an expanded library of compounds and can also serve different applications in both biological and electronic systems.

### 2.2. X-ray Crystallography

**Figure 2:**
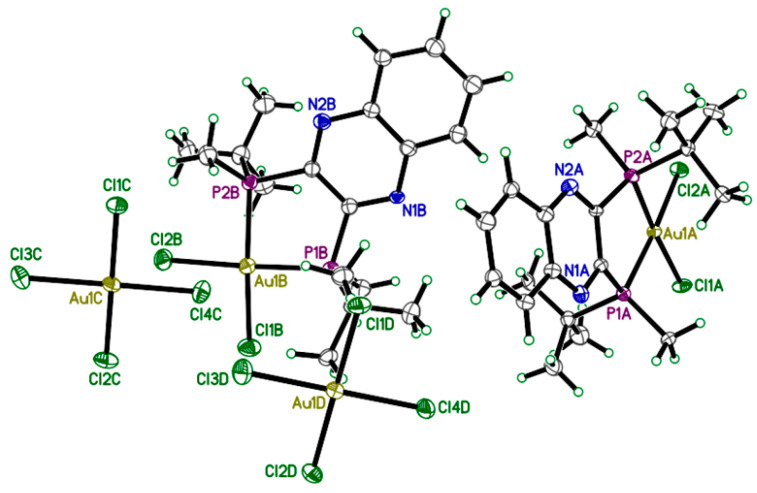
X-ray crystal structure of complex **2**. Depicted above is the full unit cell; two cations and two tetrachloroaurate(III) anions. All atoms are drawn at 50% probability level. Hydrogen atoms have been omitted for clarity.

Crystals of **2** were grown by slow diffusion of ether into a concentrated DCM solution of gold complex and analyzed by X-ray diffraction. The unique structure of **2** which occurs as the *S*,*S*-QuinoxP* ligand reacts with two molar equivalents of HAuCl_4_·3H_2_O (Scheme 1) crystallizes as a monocationic complex in a triclinic crystal system with P1 space group (Figure 2). The X-ray structure of **2** reveals a C_2_-symmetric square-planar geometry around the gold centre, with two t-butylmethyl phosphine groups from the QuinoxP* ligand and two chloride ligands from HAuCl_4_·3H_2_O defining the *d^8^* Au(III) geometry, as shown in Figure 2. The bond length (Table 1) of the Au-P bond is Au1A-P2A: 2.292(6) Å and Au1A-P1A: 2.298(6) Å, while the Au-Cl bond is Au1A-Cl2A: 2.320(6) Å and Au1A-Cl1A: 2.327(6) Å. The bond angle for the P2A-Au1A-P1A is 89.4(2)° and Cl2A-Au1A-Cl1A is 91.8(2), indicating a square planar geometry. The stereochemistry was determined by placing the ellipsoid diagram of complex 2 in the plane using the Mercury program [41]. The X-ray Parameters of **2** is shown in Table 2.

### 2.3. Biological Stability of ***1*** and ***2***

#### 2.3.1. UV–Vis Stability in Biological Media

**Figure 3:**
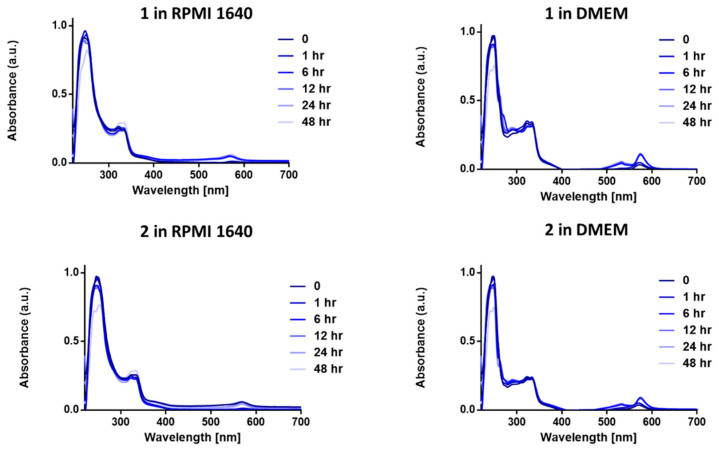
UV−vis spectra of **1** and **2** (50 μM) in Dulbecco’s modified eagle medium (DMEM) and Roswell Park Memorial Institute-1640 (RPMI-1640) over a period of 48 h showing the stability of compounds **1** and **2** at 37 °C.

We first carried out stability studies on **1** and **2** in two biological media—Dulbecco’s modified eagle medium (DMEM) and Roswell Park Memorial Institute-1640 (RPMI-1640); both DMEM and RPMI-1640 contains large amount of amino acids, glucose, and other biological nucleophiles. This is important as anticancer drugs should be stable and not decompose before reaching their intracellular targets. Gold complexes, in particular Au(III) complexes, are highly susceptible to reduction by biological nucleophiles [42,43,44], especially cysteine containing residues. The stability of complexes in biological medium is pertinent to the efficacy and longevity of the chemotherapeutic in question [44]. The two complexes (**1** and **2**) were observed for a period of 48 h in biological buffer solutions at 37 °C to mimic the body temperature. Both **1** and **2** exhibited great stability in both DMEM and RPMI-1640 over the course of the experiment (Figure 3). Given that most complexes are cleared within 60–80 min [45], the relative high stability of these complexes makes them a great framework for gold(III) chemotherapeutic development. The high-energy bands and intense absorption observed in the UV–vis spectra of **1** and **2** in RPMI at 247 and 322 nm may be attributed to the ligand-to-metal charge transfer (LMCT) or metal-to-ligand charge-transfer (MLCT) character of the Au(III) center. There was no change in the absorption profile of **1** or **2** in RPMI 1640 and a slight change in DMEM. The stability of **1** and **2** in RPMI can be attributed to the Au-P bonds that enhance sigma-donation to the gold center. The slight hump at 568 nm in the spectra of **1** and **2** in DMEM can be attributed to displacement of the chloride in **1** and **2** by some amino acids in DMEM.

#### 2.3.2. Reactivity of **1** and **2** with l-Glutathione (GSH)

Next, we monitored the reaction of **1** and **2** with cysteine thiols using *L*-Glutathione (GSH) at different concentrations (0, 50 and 500 µM) via UV–vis spectrophotometry. Reactions of gold complexes with amino acids and other biological nucleophiles are well documented [43,46,47,48]. For example, organogold(III) compounds have been found to undergo reduction at the gold center when reacted with protein hen egg white lysozyme (HEWL) [43]. Additionally, activation of the gold center is believed to take place via the displacement of the labile chloride atom without alteration of the overall complex [47]. Further, Au(III) dithiocarbamates have also been studied and they are rapidly reduced in the presence of biological nucleophiles, which limit their application [49,50].

**Figure 4:**
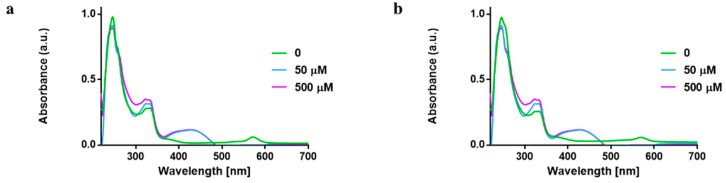
UV–vis spectra showing reaction of *L*-glutathione with (**a**) **1** and (**b**) **2** in RPMI at 37 °C at different concentration.

Reaction of **1** and **2** with GSH in ratio 1:1 or 1:10 did not alter the absorption bands associated with the gold complexes, suggestive of high compound stability in GSH. The UV–vis absorption band at 242 and 330 nm may be as a result of ligand-to-metal charge transfer (LMCT) or metal-to-ligand charge-transfer (MLCT). Additionally, there is also a possibility of the displacement of the chloride ions by GSH in **1** and **2**, which is seen at a higher concentration with the absorption band at 430 nm, yet it is clear that the compound is not reduced to elemental gold in the biological media (Figure 4. The solution studies suggest that **1** and **2** are not decomposed in biological nucleophiles and hence they could reach their target cells without premature deactivation in cells.

### 2.4. Cytotoxicity of ***1*** and ***2***

**Figure 5:**
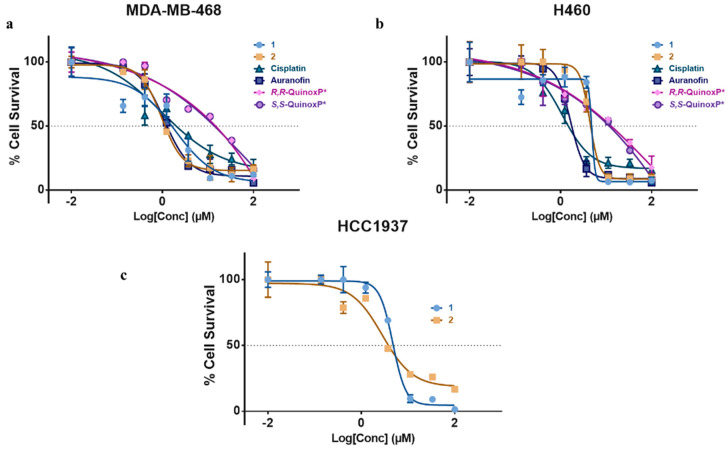
In vitro activity of complexes **1** and **2**, *R*,*R*-QuinoxP* and *S*,*S*-QuinoxP*, in (**a**) MDA-MB-248, (**b**) H460 and (**c**) HCC1937 cancer cell lines showing IC_50_ plot for complexes after 72 h. Cell survival was determined with crystal violet assay. Data are plotted as the mean ± s.e.m (n = 3).

The antiproliferative activity of the two complexes was compared against the ligands (*R*,*R*-QuinoxP* and *S*,*S*-QuinoxP*, auranofin, cisplatin by monitoring the inhibition of cell growth using a crystal violet assay. The cytotoxic activity was determined in three human cancer cell lines—MDA-MB-468 triple negative breast cancer cell (TNBC), HCC1937 breast cancer cell line (TNBC) and H460 lung cancer cell line. The IC_50_ values were extrapolated from dose–response curves, and these results have been summarized in Table 3 and Figure 5. Compounds **1** and **2** were cytotoxic in all the cell lines studied. They exhibited higher cytotoxicity in the TNBC cell line, MDA-MB-468 studied with an IC_50_ of 1.51 and 1.08 µM compared to their respective ligands, auranofin and cisplatin. The cytotoxicity of **1** and **2** in the lung cancer cell line (H460) was slightly lower than that of cisplatin and auranofin. The cytotoxicity observed in these complexes makes them possible candidates as gold-based anticancer agents. It is noteworthy to say that the two novel complexes do not show a marked difference in cytotoxicity compared to other enantiomeric complexes.

### 2.5. Electrochemical Studies of ***2***

**Figure 6:**
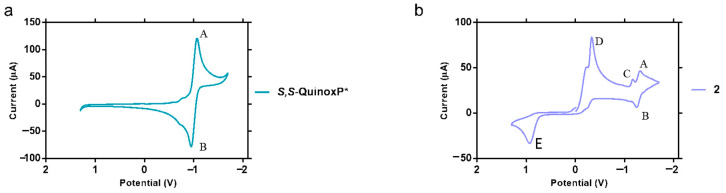
Cyclic voltammograms of (**a**) *S*,*S*-QuinoxP* ligand and (**b**) complex **2**. This experiment was performed at room temperature in acetonitrile solution with 0.10 M NBu_4_PF_6_ electrolyte at 100 mV/s scan rate. The potential is referenced to Ag/AgCl.

The electrochemical behaviour of complex **2** was characterized by cyclic voltammetry in acetonitrile with 0.1M NBu_4_PF_6_ as the supporting electrolyte. The electrochemical parameters of **2** and *S*,*S*-QuinoxP* are shown in Table 3, and the voltammograms are shown in Figure 6. The condition used was a scan rate of 100 mV/s referenced to Ag/AgCl. The cyclic voltammogram of **2** exhibited features different from the ligand. Complex **2** shows a reversible reduction at a potential at A and B (−1.32 and −1.25 V) which can be attributed to the QuinoxP* ligand as the free ligand also undergoes a reversible one electron reduction at −1.06 and −0.95 V (Figure 6a). Compound **2** also showed an irreversible reduction at C (−1.16 V) corresponding to Au(III)/Au(I). The peak at D (−0.33 V) is attributed to the reduction in P(III)/(I) due to the coordination of P to the metal center [52]. Without further electro-spectrophotometry, it is unclear what the oxidation wave of **2** at E (0.93 V) is (Figure 6b).

## 3. Materials and Methods

### 3.1. General Experimental Details

All reactions were carried out under ambient conditions in air unless otherwise noted. Solvents were of ACS grade (Pharmco-Aaper) and used as is. HAuCl_4_·3H_2_O was purchased from Nano Partz and stored under nitrogen atmosphere. *R*,*R*-QuinoxP* and *S*,*S*-QuinoxP* were purchased from Strem Chemicals. Deuterated solvents were purchased from Cambridge Isotope Laboratories (Andover, MA, USA). NMR spectra were recorded on a Bruker Avance NEO 400 MHz spectrometer and samples calibrated for: ^1^H NMR (CDCl_3_ δ = 7.26 ppm), ^13^C NMR (CDCl_3_ δ = 77.16) and ^31^P NMR externally referenced to H_3_PO_4_ *δ* = 0.00. High-resolution mass spectra (HRMS) were obtained by direct flow injection (injection volume = 2 μL) using ElectroSpray Ionization (ESI) on a Waters Synapt G2 HDMS instrument in the positive mode with a quadripole/TOF analyzer (UC Boulder). Elemental analysis results were obtained from Atlantic Microlabs, Inc (Norcross, GA, USA). In addition to spectroscopic characterization, the purity of all compounds was assessed by reverse phase high performance liquid chromatography (RP-HPLC) using an Agilent Technologies 1100 series HPLC instrument and an Agilent Phase Eclipse Plus C18 column (4.6 × 100 mm; 3.5 µm particle size). All compounds were found to be ≥ 97% pure.

### 3.2. Synthesis of Compounds ***1*** and ***2***

Synthesis of [((*R*,*R*)-(–)-2,3-Bis(tert-butylmethylphosphino)quinoxaline)AuCl_2_][AuCl_4_] (**1**): HAuCl_4_·3H_2_O (91.87 mg, 0.233 mmol) was dissolved in DCM (2 mL) and *R*,*R*-[2,3-bis(tert-butylmethylphosphino)quinoxaline (39 mg, 0.117 mmol) was dissolved in 2 mL of DCM which was added dropwise. The reaction mixture turned red while adding and the mixture was left to stir at room temperature until the color changed to light orange after 45 min. The reaction was filtered through celite, concentrated in vacuo, and the compound precipitated with ether to yield a yellow colored solid. Yield: 40 mg, (36.4%). ^1^H NMR (400 MHz, CDCl_3_) 8.55 (dd, *J* = 6.4, 3.6 Hz, 2H), 8.31 (dd*, J* = 6.8, 3.6 Hz, 2H), 2.79 (d, *J* = 16 Hz, 6H), 1.51 (d, *J* = 20 Hz, 18H); ^13^C NMR (101 MHz, CDCl_3_) δ = 146.46, 137.11, 130.97, 65.87, 28.23, 15.29; ^31^P NMR (162 MHz, DMSO-d_6_) δ = 70.28. HRMS *(m/z)* calcd. 601.0770, found 601.0781 [M − AuCl_4_]^+^ (Figures S7 and S8). Elemental analysis calcd. %C 22.97, %H 3.00, %N 2.98; found %C 23.24, %H 3.07, %N 3.10. Purity was determined to be >97% by RP-HPLC: R_f_ = 9.33 min using the following method: Flow rate: 1 mL/min; λ = 280 nm; Eluent A = H_2_O with 0.1% TFA; Eluent B = MeCN with 0.1% Formic acid; Solvent Gradient: 0–7 min (70:30 H_2_O:MeCN), 5 min (50:50 H_2_O:MeCN), 10 min (100 MeCN) and 15 min (20:80 H_2_O:MeCN).

Synthesis of [((*S*,*S*)-(+)-2,3-Bis(tert-butylmethylphosphino)quinoxaline)AuCl_2_][AuCl_4_] (**2**): HAuCl_4_·3H_2_O (91.87 mg, 0.233 mmol) was dissolved in DCM (2 mL) and *S*,*S*-[2,3-bis(tert-butylmethylphosphino)quinoxaline (37 mg, 0.111 mmol) dissolved in 2 mL of DCM was added dropwise, the reaction mixture turned red while adding and the mixture was left to stir at room temperature until the color changed to light orange after 45 min. The reaction was filtered through celite, concentrated in vacuo, and the compound precipitated with ether to yield a yellow colored solid. Yield: 40 mg, 41.5%). ^1^H NMR (400 MHz, CDCl_3_) 8.54 (dd, *J* = 8, 3.2 Hz, 2H), 8.29 (dd, *J* = 6, 2.8, Hz, 2H), 2.77 (d, *J* = 16 Hz, 6H), 1.48 (d, *J* = 20 Hz, 18H); ^13^C NMR (101 MHz,CDCl_3_) δ = 143.25, 137.03, 130.34, 41.38, 28.08, 7.72; ^31^P NMR (162 MHz, DMSO-d_6_) δ = 70.45. HRMS *(m/z)* calcd. 601.0770, found 601.0781 [M – AuCl_4_]^+^ (Figures S11 and S12). Elemental analysis (%) calcd. %C 22.97, %H 3.00, %N 2.98; Found %C 23.12, %H 3.01, %N 3.04. Purity was demonstrated to be >97% by RP-HPLC: R_f_ = 10.4 min using the following method: Flow rate: 1 mL/min; λ = 280 nm; Eluent A = H_2_O with 0.1% TFA; Eluent B = MeCN with 0.1% Formic acid; Solvent Gradient: 0–7 min (70:30 H_2_O:MeCN), 5 min (50:50 H_2_O:MeCN), 10 min (100 MeCN) and 15 min (20:80 H_2_O:MeCN).

### 3.3. Physical and Chemical Characterization

#### 3.3.1. X-ray Crystallography

Crystals of complex **2** were grown from slow diffusion of Et_2_O into a concentrated solution of MeCN at room temperature. All crystals were mounted using polyisobutene oil on the end of a glass fiber, which had been mounted to a copper pin using an electrical solder. It was placed directly in the cold gas stream of a liquid nitrogen cryostat [53,54]. A Bruker D8 Venture diffractometer with graded multilayer focused MoKα X-rays (λ = 0.71073 Å) was used to collect diffraction. Raw data were integrated, scaled, merged and corrected for Lorentz-polarization effects using the APEX3 package [55,56,57]. Space group determination and structure solution and refinement were carried out with SHELXT and SHELXL, respectively [58,59]. All non-hydrogen atoms were refined with anisotropic displacement parameters. Hydrogen atoms were placed at calculated positions and refined using a riding model with their isotropic displacement parameters (Uiso) set to either 1.2Uiso or 1.5Uiso of the atom to which they were attached. Ellipsoid plots were drawn using SHELXTL-XP [60]. The structures, deposited in the Cambridge Structural Database, were checked for missed symmetry, twinning and overall quality with PLATON, [61] an R-tensor, [62] and finally validated using CheckCIF [61].

#### 3.3.2. UV–Vis Stability

DMEM and RPMI-1640 were purchased from Corning Inc. and used without further supplements. Spectra were recorded on a Shimadzu-1280 spectrophotometer. Prior to use, the media were warmed to 37 °C and incubated at that temperature throughout the course of the experiment. The complexes were prepared as 1 mM stock in DMSO and subsequently diluted to a final concentration of 50 µM in each respective medium. No precipitation of colloidal gold was observed. During the course of the experiment, the gold containing solutions were kept in an incubator at 37 °C to mimic biological conditions. Prior to each scan, the instrument was blanked with the corresponding medium. The UV–vis spectra were then recorded at various time intervals (total time = 48 h) to compare longevity of the complex.

#### 3.3.3. Cyclic Voltammetry of **2**

Electrochemical measurements of the ligands were recorded with a scan rate of 0.1 V/s with a three-segment sweep and a sample interval of 0.001 V. The quiet time was set to 2 s and sensitivity and 1 × 10^−4^ A/V. All solutions were freshly prepared prior to use. All spectra were recorded using a CH instruments 650E potentiostat. The electrodes used were all 3 mm: glassy carbon working electrode (CHI104), Ag/AgCl reference electrode (CHI111) and a platinum wire counter electrode (CHI115). Compound **2** as well as *S,S*-[2,3-bis(tert-butylmethylphosphino)quinoxaline] were prepared as a 5 mM solution in dry MeCN with NBu_4_PF_6_ (0.1 M) as the supporting electrolyte. The samples were purged with nitrogen for 30 min and recorded. Data were analyzed with GraphPad Prism6.

### 3.4. In Vitro Biological Assays

#### 3.4.1. Cell Culture

All cell lines were purchased from ATCC and routinely grown in a humidified incubator at 37 °C with 5–10% CO_2_. MDA-MB-468 were grown in DMEM supplemented with 10% FBS, 1% amphotericin and 1% penicillin/streptomycin. H460, and HCC1937 cells were grown in RPMI supplemented with 10% FBS, 1% amphotericin and 1% penicillin/streptomycin, as well as 4 mM glutamine. All supplements along with PBS and trypsin-ethylenediaminetetraacetic acid (trypsin-EDTA) were purchased from Corning Inc. and used as is.

#### 3.4.2. Cell Viability of **1** and **2**

The cell viabilities of **1** and **2**, were determined in MDA-MB-468, H460 and HCC1937. Additionally, cisplatin, auranofin and both *S*,*S* and *R*,*R*-QuinoxP* were determined in MDA-MB-468 and H460. Cells were grown to confluency and trypsin was added to detach and harvest cells. The cells were washed with 2 mL of PBS and suspended in 10 mL of the appropriate media. The cells were centrifuged at 2000 rpm for 5 min and the pellet washed with 2 mL of PBS then suspended in 5 mL of the appropriate media. The cells were plated at a density of 2000 cells/well in a 96-well clear bottom plate and allowed to adhere overnight at 37 °C with 5–10% CO_2_. The compounds were prepared as a stock in DMSO and used fresh. The compounds were added at seven concentrations with a 3× serial dilution starting at 50 µM for the highest concentration and incubated at 37 °C for 72 h with 5–10% CO_2_. The cells were fixed with 1% glutaraldehyde (in PBS) and staining with 0.5% crystal violet, as previously described [15]. The plates were read using a Genios plate reader (λ = 570 nm). The experiment was performed in triplicate and data are plotted as the mean ± s.e.m. (*n* = 3).

#### 3.4.3. Reactivity of **1** and **2** with L-GSH

Interactions between **1** and **2** were performed via monitoring with UV–vis spectrometry on a Shimadzu-1280 spectrophotometer. Solutions of **1** and **2** were prepared similarly to the stability studies. A 50 µM solution of both compounds were prepared in RPMI-1640 that was pre-warmed to 37 °C. The compounds were run alone and recorded as a concentration 0 µM addition of L-GSH. L-GSH was then added portion-wise to the solution to achieve 50 µM and the spectrum recorded. L-GSH was added portion-wise to achieve a concentration of 500 µM and the subsequent spectrum was recorded.

## 4. Conclusions

Two new compounds (*R*,*R*)-(–)-2,3-Bis(*tert*-butylmethylphosphino)quinoxalinedichlorogold(III) tetrachloroaurates(III) and (*S*,*S*)-(+)-2,3-Bis(*tert*-butylmethylphosphino)quinoxalinedichlorogold(III) tetrachloroaurates(III) were synthesized and found to be cytotoxic in three cancer cell lines tested. With an IC_50_ of 1.51 µM and 1.08 µM for **1** and **2** in MDA-MB-468 triple negative breast cancer cell line, these compounds compare well with cisplatin (IC_50_ = 2.09 µM) and auranofin (1.312 µM). Their cytotoxicity in H460 and HCC1937 is slightly higher than that of cisplatin and auranofin. The X-ray crystal structure of **2** shows that the geometry around the gold center is square planar and the electrochemical studies and reaction with GSH shows that the compounds are stable as it does not decompose within the period under investigation. The stability of the two compounds in biological media and promising antiproliferative potential places these compounds as candidates for therapeutic development. Further biological characterization of **1** and **2** needs to be carried out to investigate their selectivity to normal cells and potential biological target(s) or mechanism of action.

## Figures and Tables

**Table 1:** Selected interatomic distances (Å) and angles (deg) from the crystal structure of complex **2**, shown in Figure 2.

Selected Atom	Selected Atomic Distances (Å) and Angles (°)
Au1A-P2A	2.292(6) Å
Au1A-P1A	2.298(6) Å
Au1A-Cl2A	2.320(6) Å
Au1A-Cl1A	2.327(6) Å
P2A-Au1A-P1A	89.4(2)°
Cl2A-Au1A-Cl1A	91.8(2)°

**Table 2:** X-ray Parameters of **2**.

X-ray Structural Data and Crystal Refinement	
Empirical Formula	C_18_H_28_Au_2_Cl_6_N_2_P_2_
Molecular Weight (g/mol)	941.00
Temperature (K)	90.0(2)
X-ray Radiation (Å)	Mo Kα (0.71073 Å)
Crystal System, Space Group	Triclinic, P1
Unit Cell Dimensions (A), (o)	a = 11.0642(4) Å alpha = 80.985(1)
	b = 11.2636(4) Å beta = 83.150(1)
	c = 12.5182(4) Å gamma = 63.674 (1)
Volume	1378.79(8) Å^3^
Z	2
Absorption Coefficient	11.336 mm^−1^
F(000)	880
Crystal Size (mm)	0.120 × 0.040 × 0.030
Theta Range	2.031 to 27.512
Completeness to Theta = 25.242	100%
F^2^	1.022
Final R indices [I > 2sigma(I)]	R1 = 0.0518, wR2 = 0.1258

**Table 3:** In vitro antiproliferative activity. IC_50_ values for **1** and **2** across a panel of cell lines. Cells were seeded at a density of 2000 cells per well and treated for 72 h. IC_50_ values are plotted as the mean ± s.e.m (*n* = 3).

IC_50_ (µM)						
	1	2	*R*,*R* QuinoxP*	*S*,*S* QuinoxP*	Cisplatin	Auranofin
MDA-MB-468	1.51 ± 0.07	1.08 ± 0.13	14.8 0 ± 1.03	14.80 ± 1.03	2.09 ± 0.19	1.31 ± 0.09
H460	4.53 ± 0.09	4.53 ± 0.06	11.75 ± 1.40	11.75 ± 1.40	1.41 ± 0.12	1.69 ± 0.21
HCC1937	4.83 ± 0.1	3.85 ± 0.08	-	-	105.98 ± 0.97 ^a^	-

^a^ = Reported IC_50_ of cisplatin in HCC1937 [51].

## Supplementary Materials

The following are available online, NMR spectra of **1** and **2** (Figures S1–S6); HRMS data of **1** and **2** (Figures S7–S14); HPLC data (Figures S15 and S16); Electrochemistry (Figure S17) and Accession Code.
