# Supplementary material for: Synthesis, Characterization, and Antiproliferative Activity of Novel Chiral [QuinoxP*AuCl2]+ Complexes
Source: Molecules. 2020 Dec 4;25(23):5735. doi: 10.3390/molecules25235735 (PMC7730091; doi:10.3390/molecules25235735)
Supplement: Supplementary file 1 [file molecules-25-05735-s001.pdf]

Supporting Information for

Synthesis, Characterization and Antiproliferative Activity of Novel Chiral [QuinoxP*AuCl$_2$]$^+$ Complexes

Adedamola S. Arojojoye,[a] R. Tyler Mertens,[a] Samuel Ofori,[a] Sean Parkin,[a] and Samuel G. Awuah[a,b*]

[a]Department of Chemistry, University of Kentucky, Lexington, Kentucky 40506

[b]Department of Pharmaceutical Sciences, College of Pharmacy, University of Kentucky, Lexington, Kentucky 40536

*awuah@uky.edu

Table of contents **Page**

Section	Page
**NMR Spectra**	**S2**
**HRMS Spectra**	**S8**
**HPLC Spectra**	**S12**
**Electrochemistry**	**S13**
**X-ray Accession Code**	**S13**

NMR Spectra:

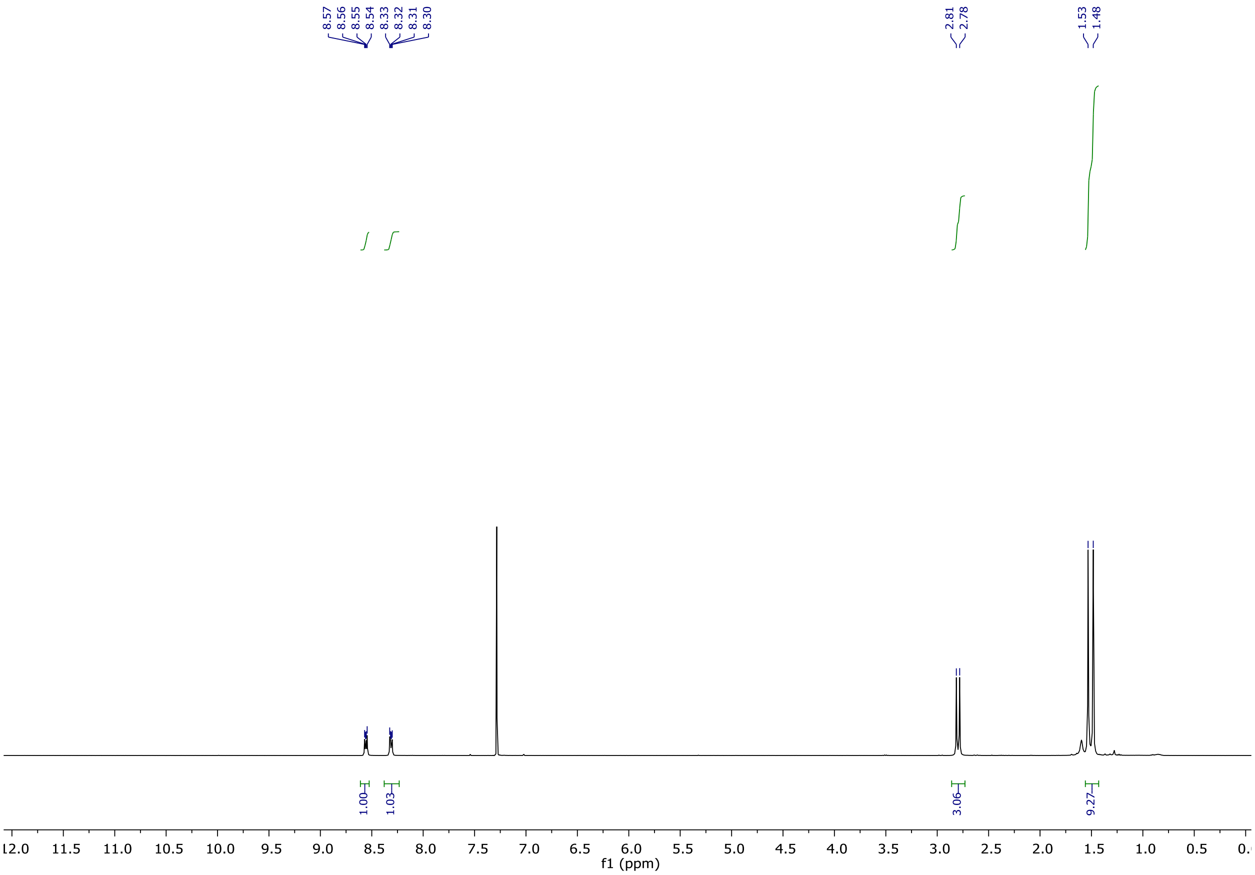

Figure S1. ^{1}H NMR spectrum of **1** in $CDCl_3$ at 298K.

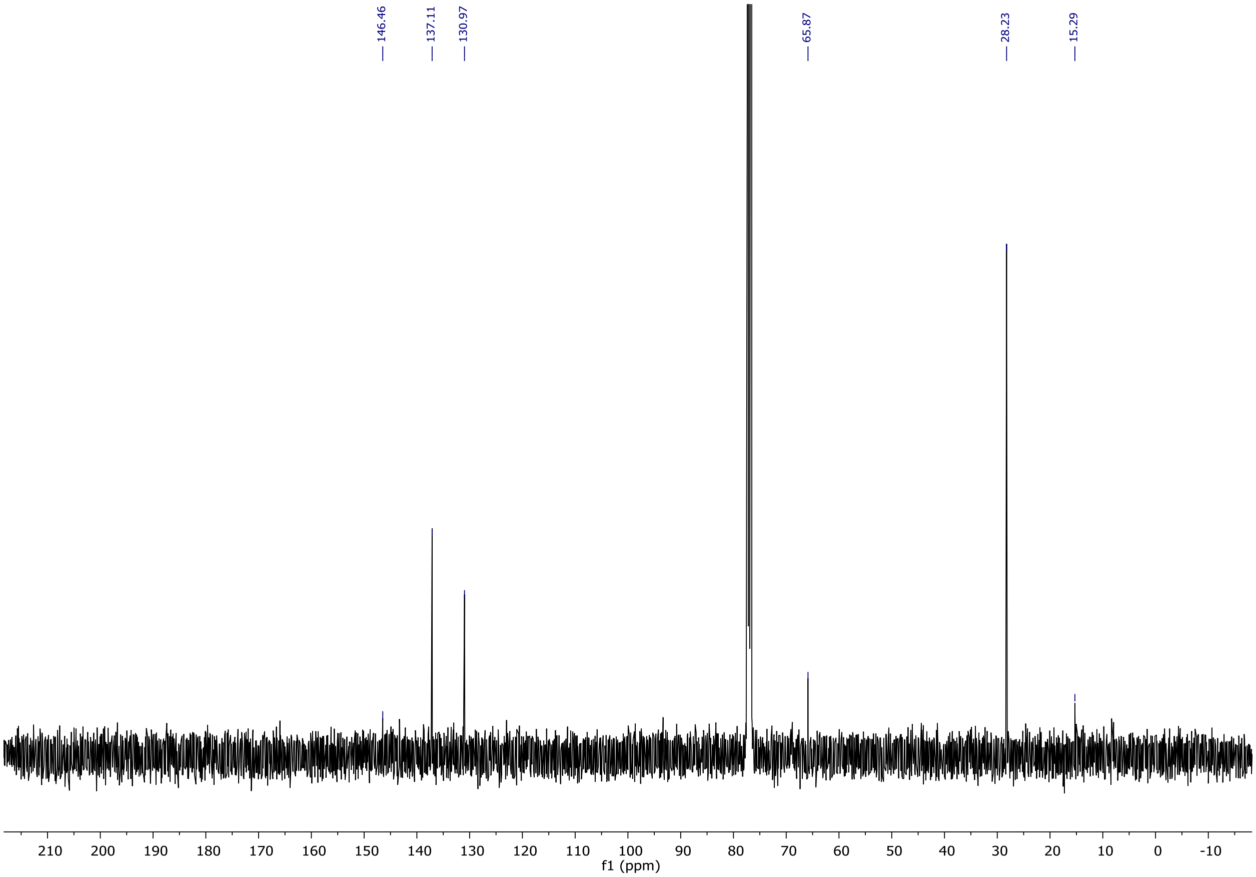

Figure S2. ^{13}C NMR spectrum of **1** in $CDCl_3$ at 298K.

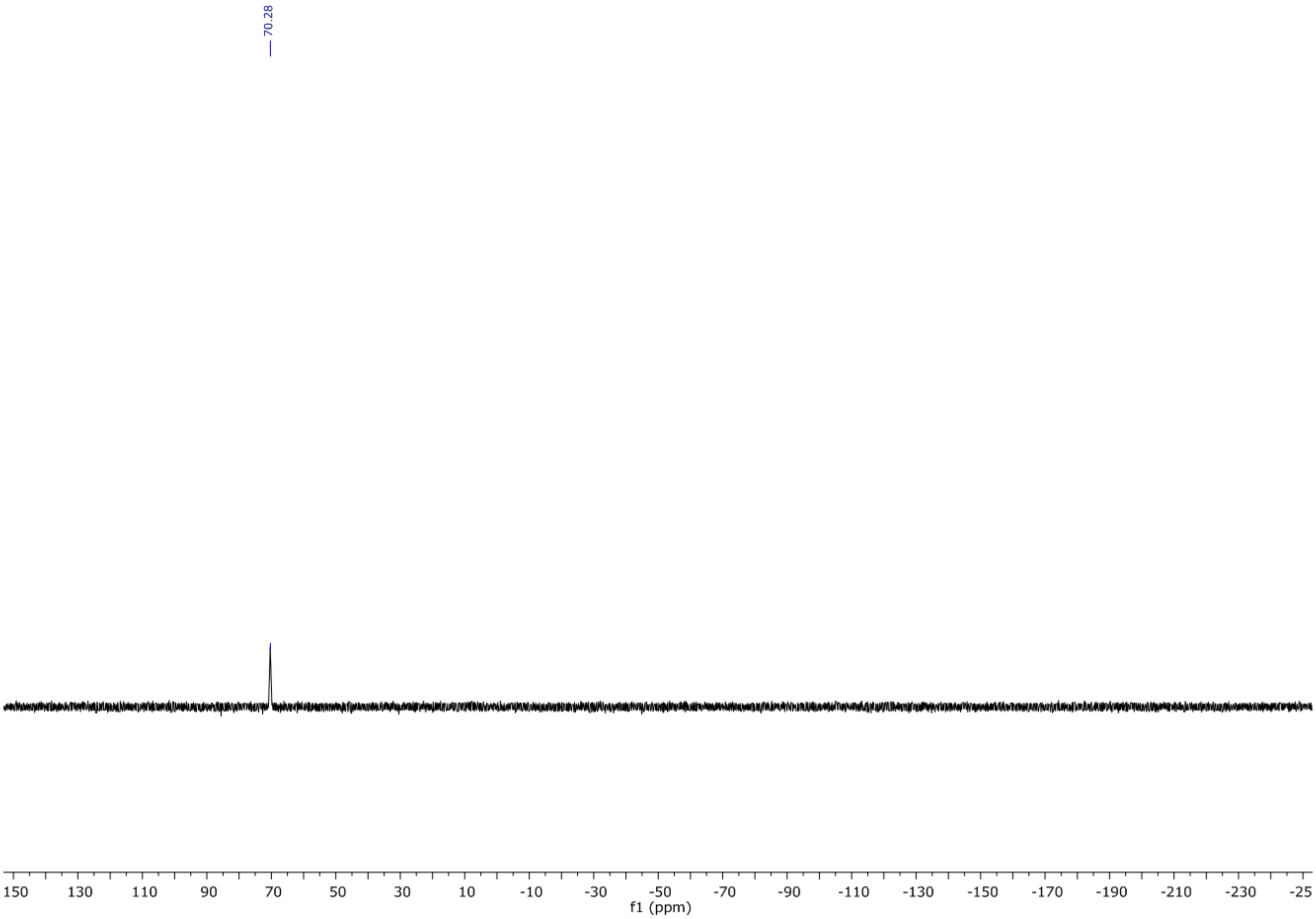

Figure S3. ^{31}P NMR spectrum of **1** in $CDCl_3$ at 298K.

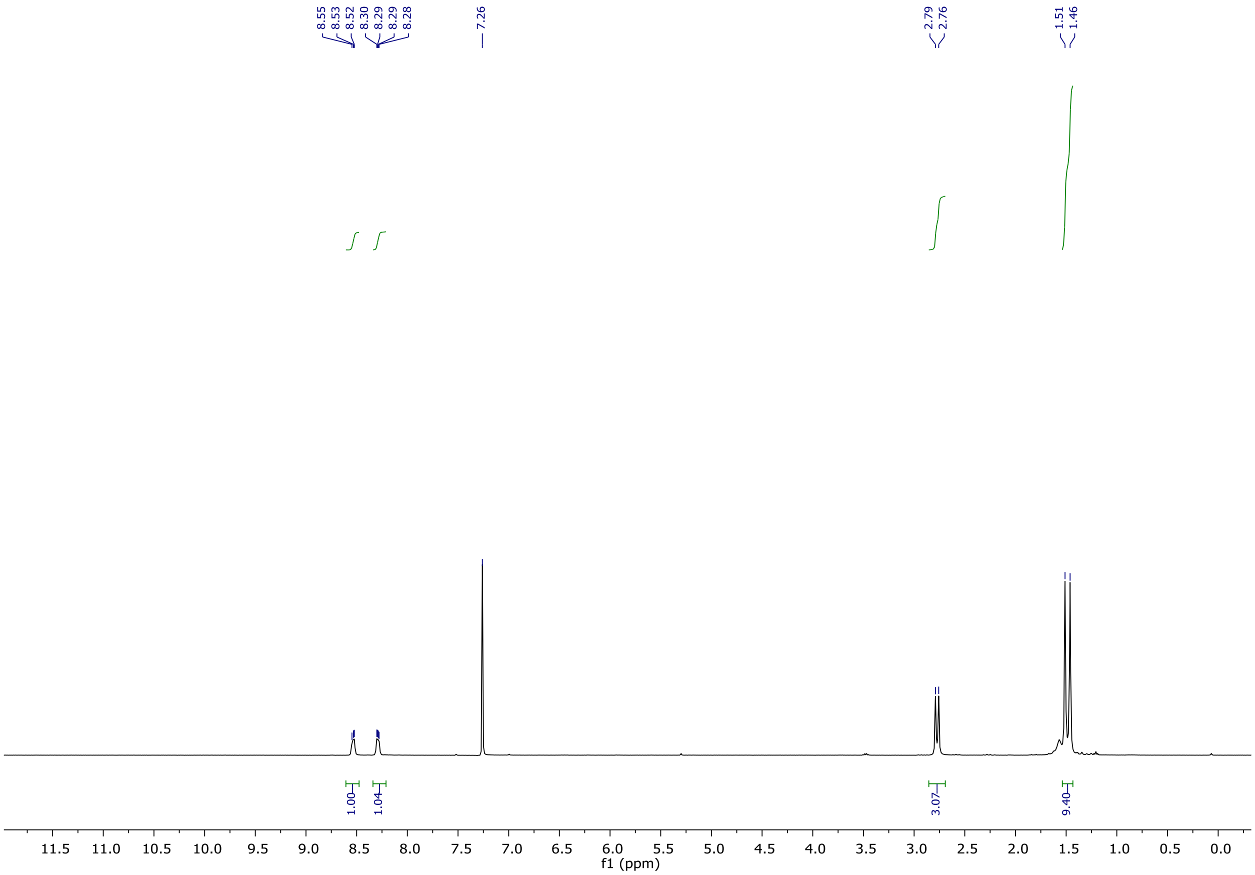

Figure S4. ^{1}H NMR spectrum of **2** in $CDCl_3$ at 298K.

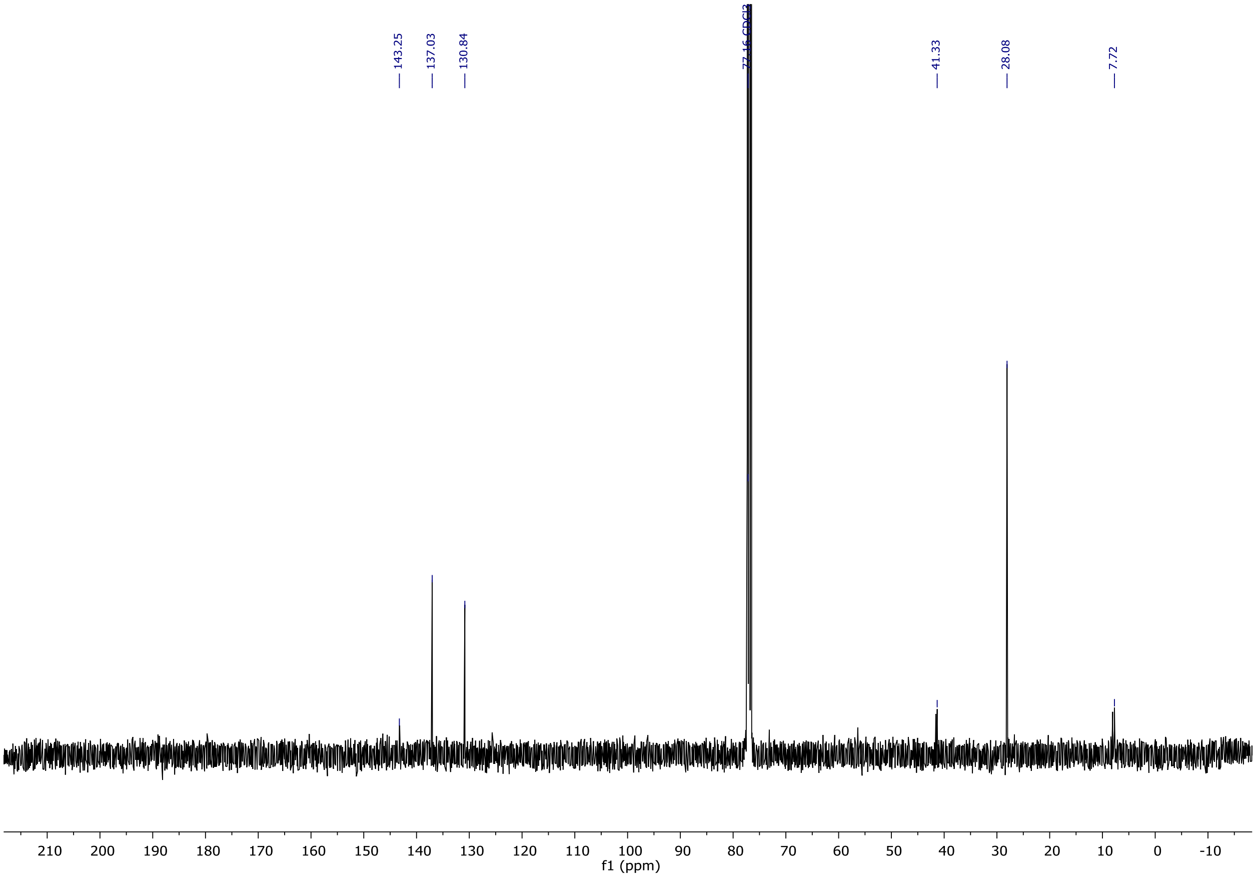

Figure. S5 ^{13}C NMR spectrum of **2** in $CDCl_3$ at 298K.

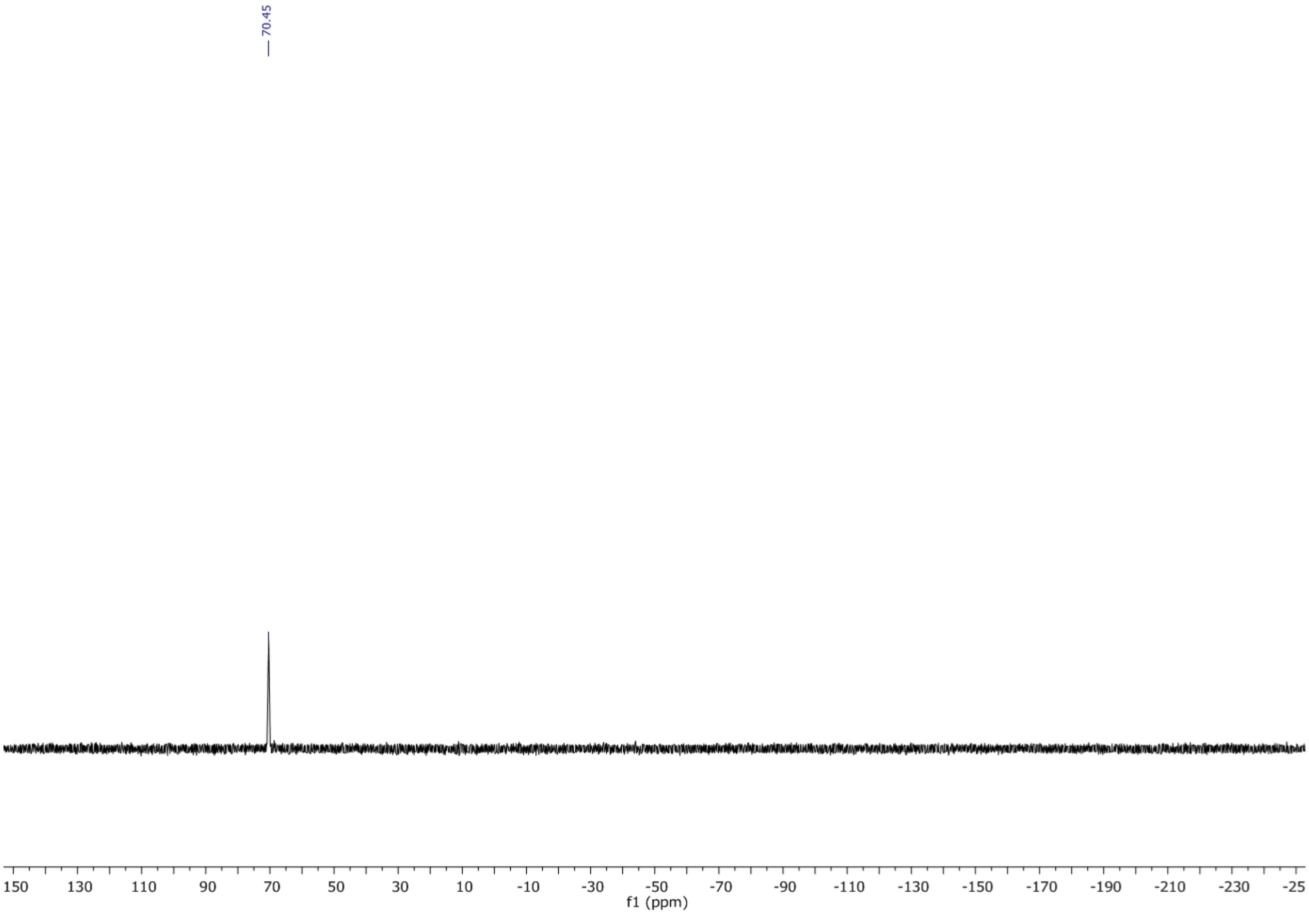

Figure. S6 ^{31}P NMR spectrum of **2** in $CDCl_3$ at 298K.

HRMS:

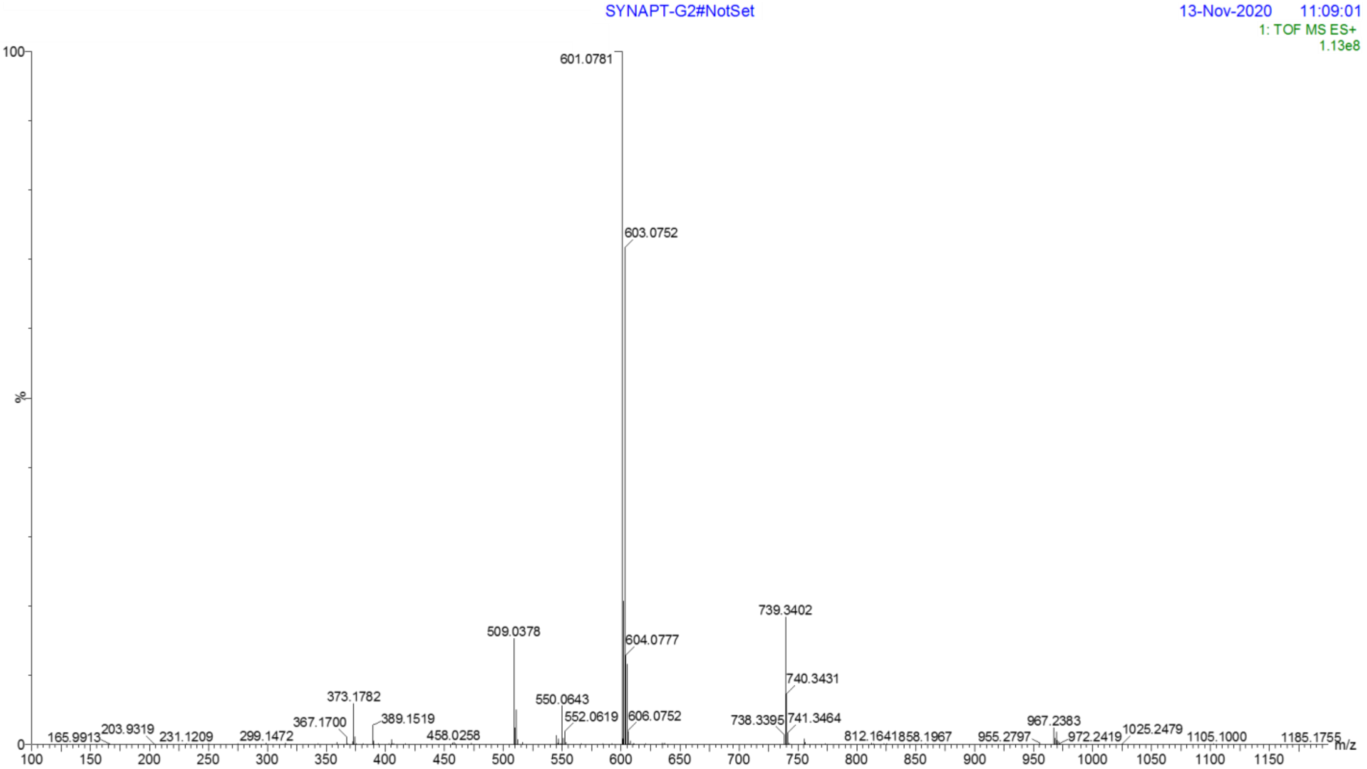

Figure S7. Full spectrum of HRMS of **1 –** TOF MS ES+

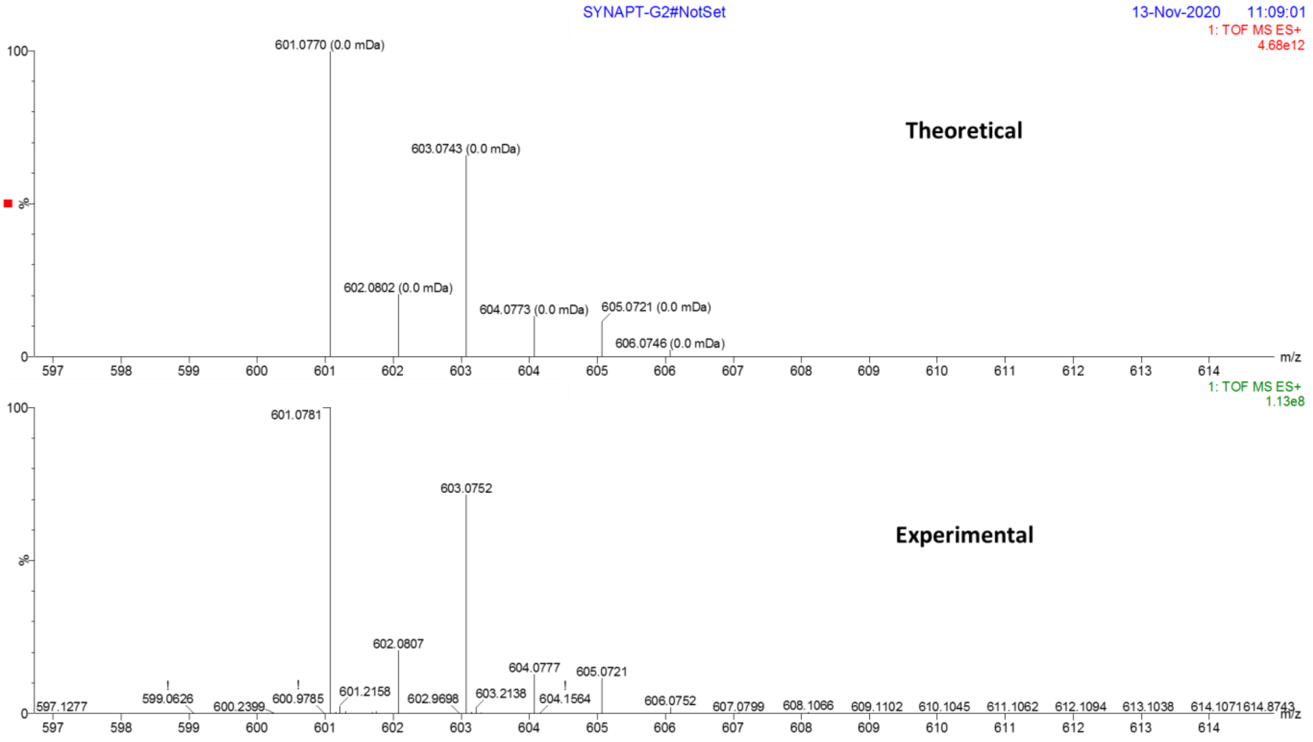

Figure S8. Experimental/Theoretical comparison of HRMS of **1 –** TOF MS ES+

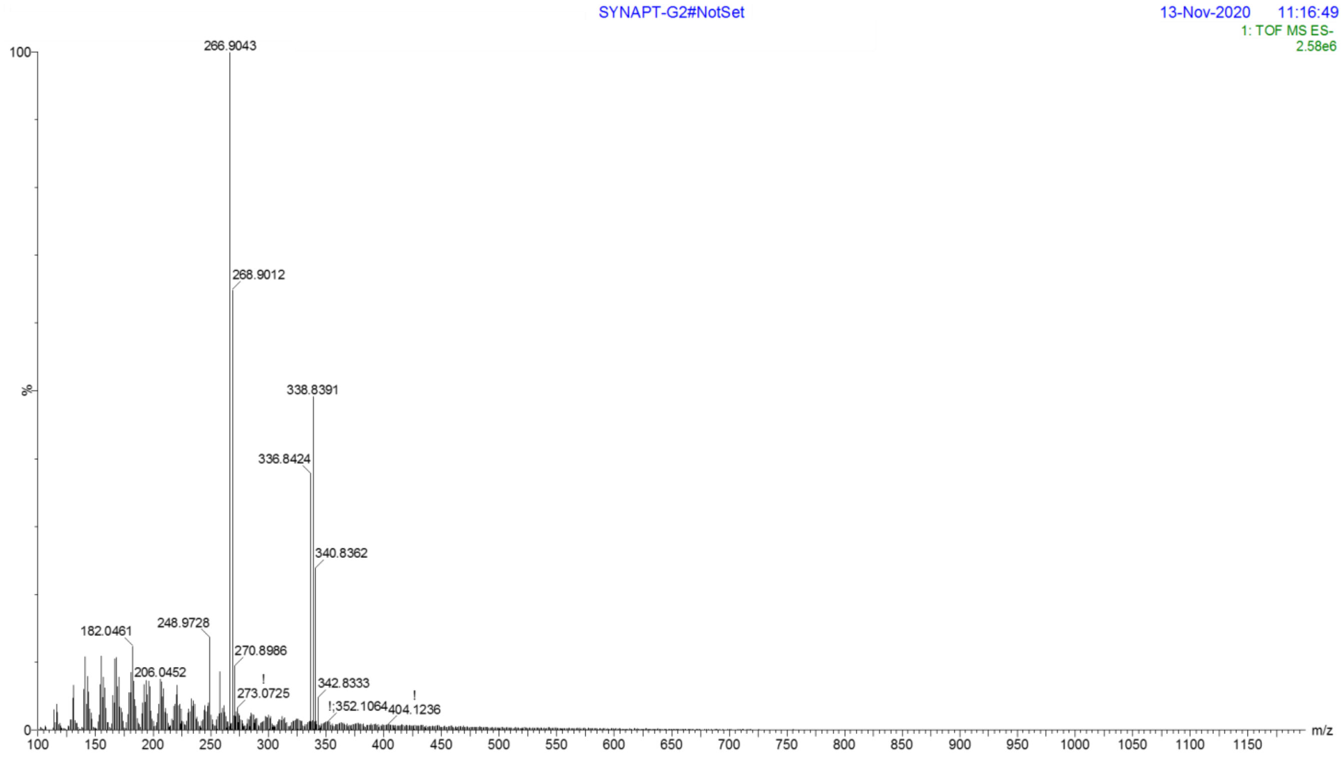

Figure S9. Full spectrum of HRMS of **1 - [AuCl₄]⁻** – TOF MS ES⁻

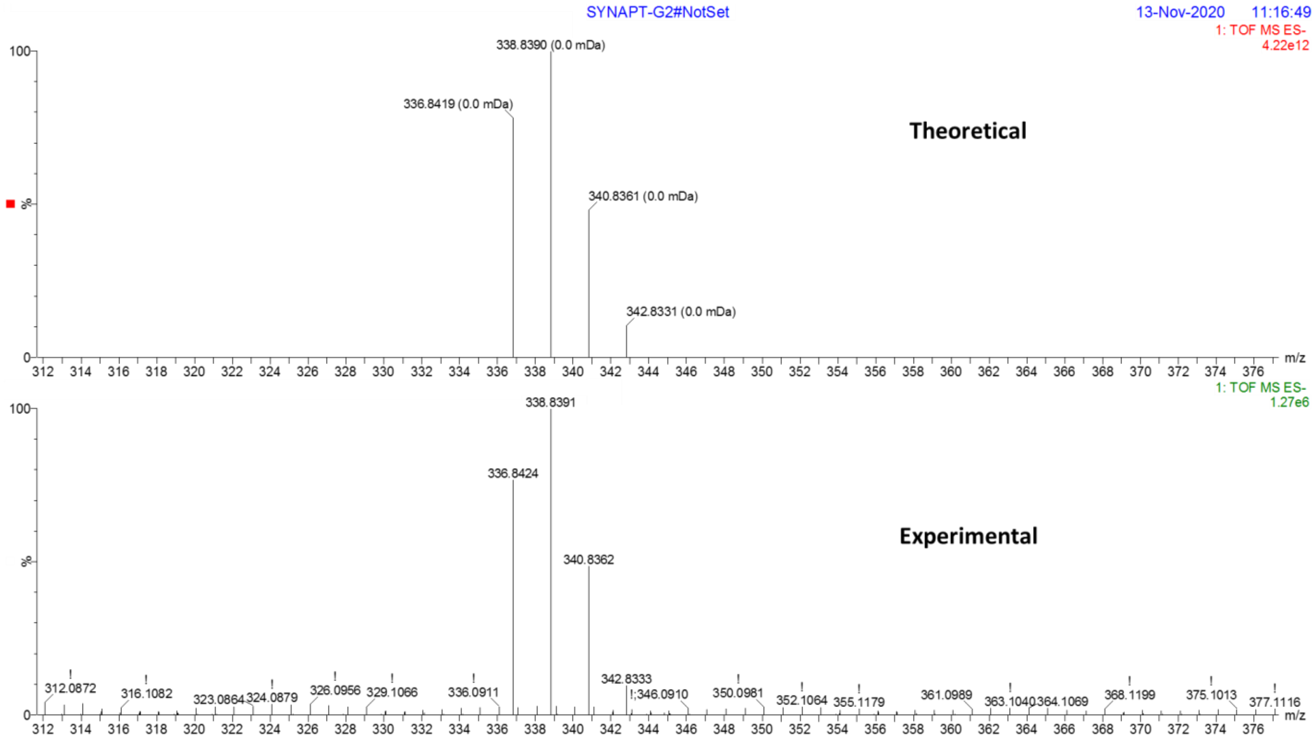

Figure S10. Experimental/Theoretical comparison of HRMS of **1 - [AuCl$_4$]$^-$ –** TOF MS ES$^-$

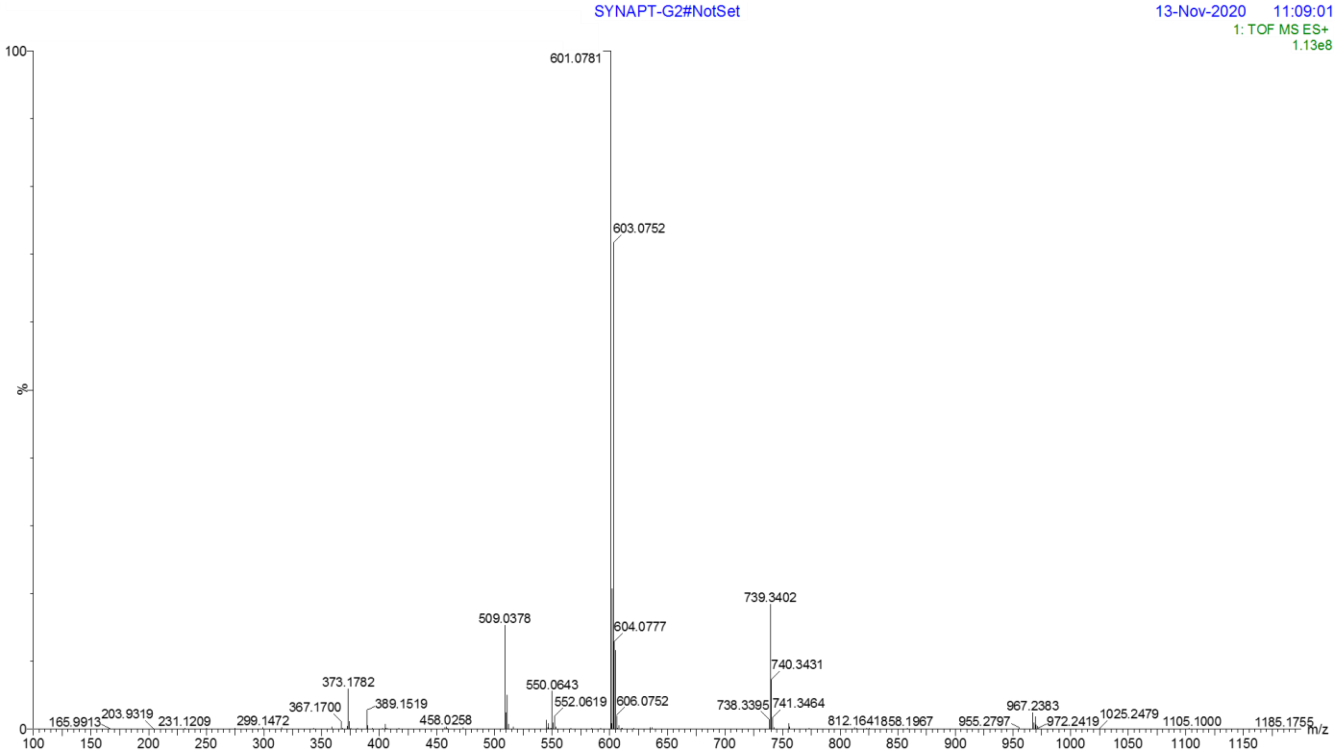

Figure S11. Full spectrum of HRMS of **2 –** TOF MS ES$^+$

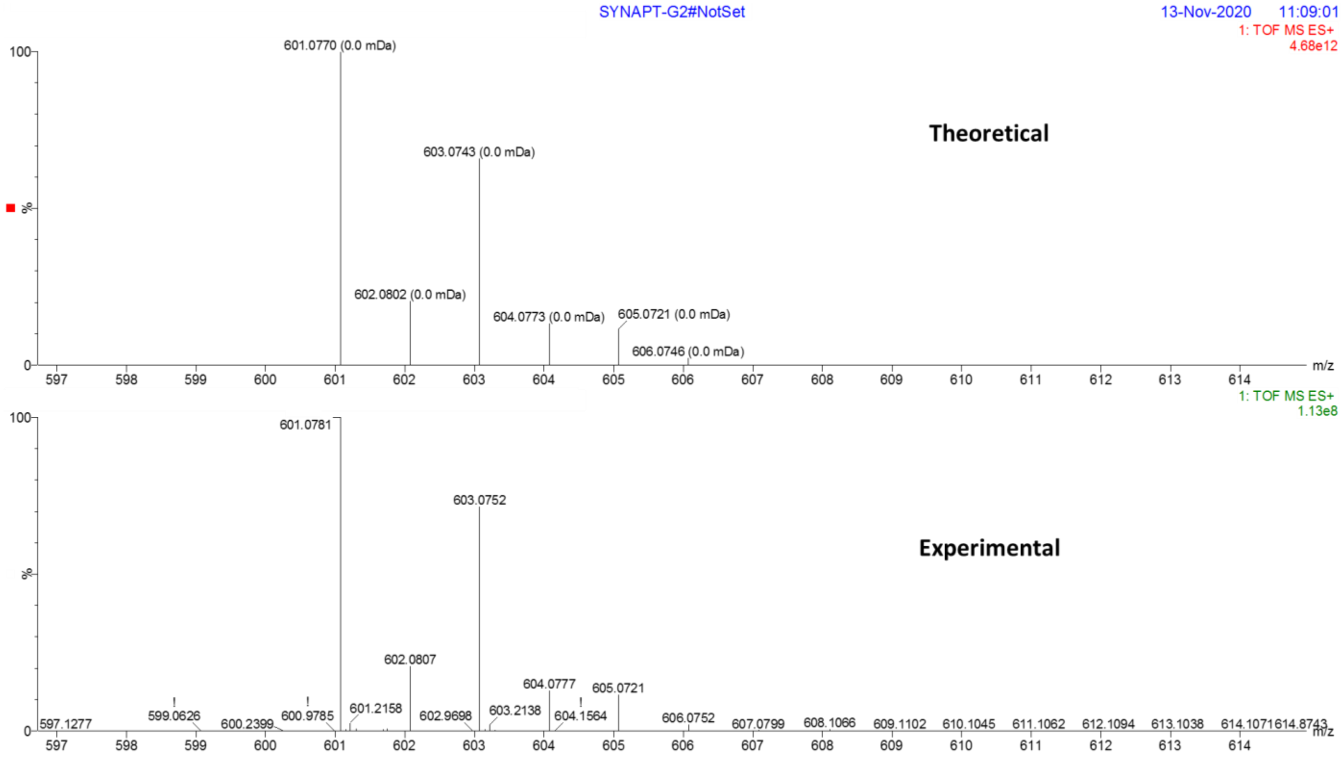

Figure. S12 Experimental/Theoretical comparison of HRMS of **2 –** TOF MS ES$^+$

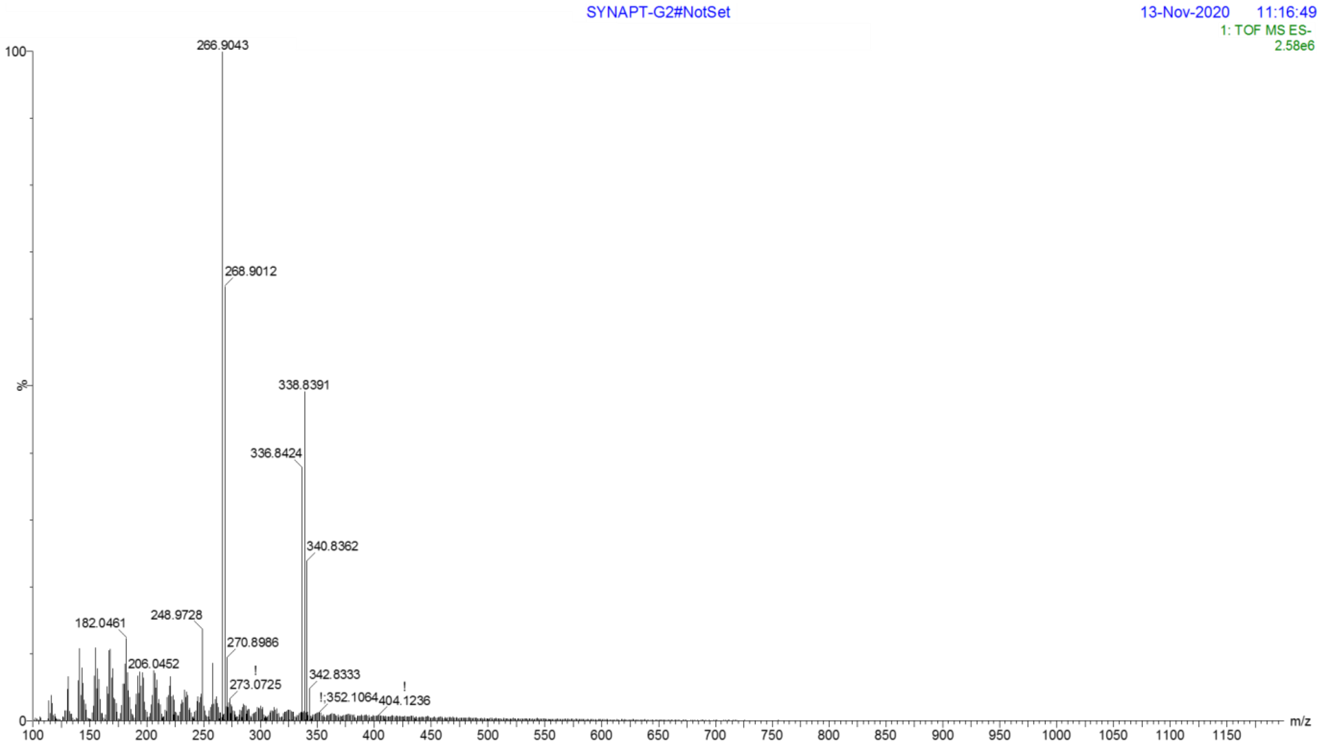

Figure. S13 Full spectrum of HRMS of **2 - [AuCl$_4$]$^-$ –** TOF MS ES$^-$

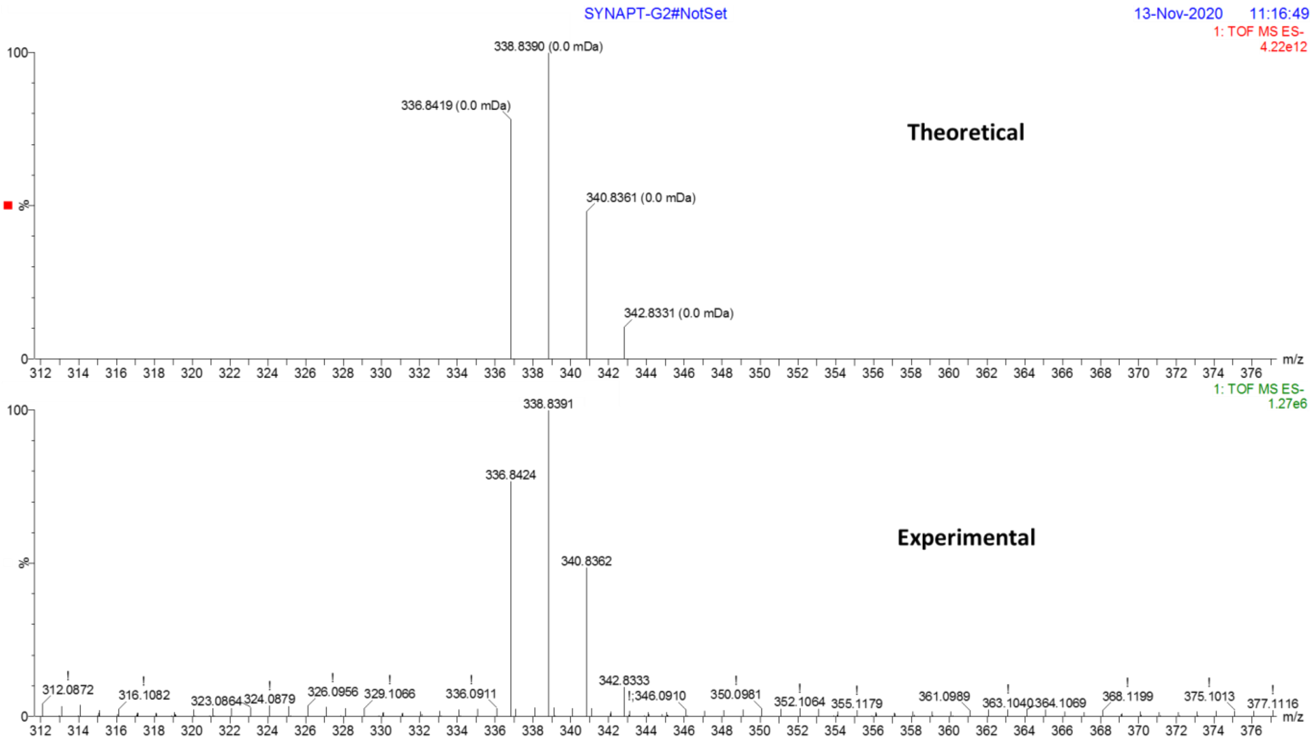

Figure S14 Experimental/Theoretical comparison of HRMS of **2 - [AuCl$_4$]$^-$ –** TOF MS ES$^-$

HPLC Trace:

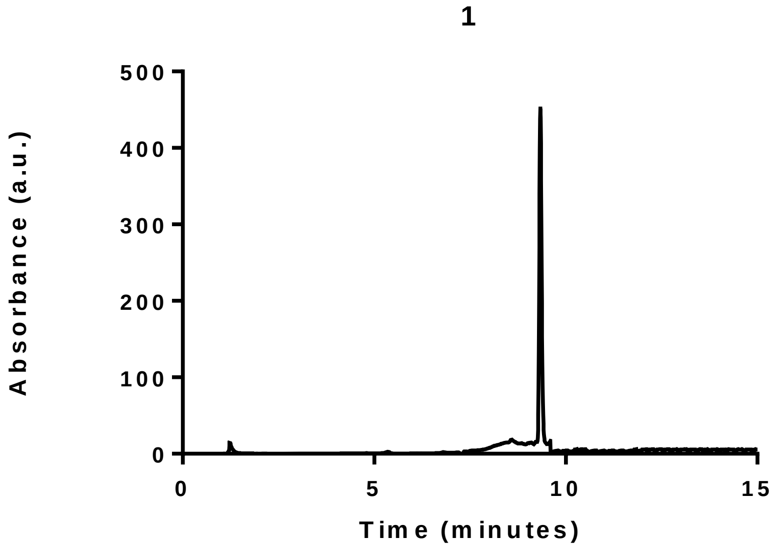

Figure S15 HPLC chromatogram of **1,** (λ = 280 nm).

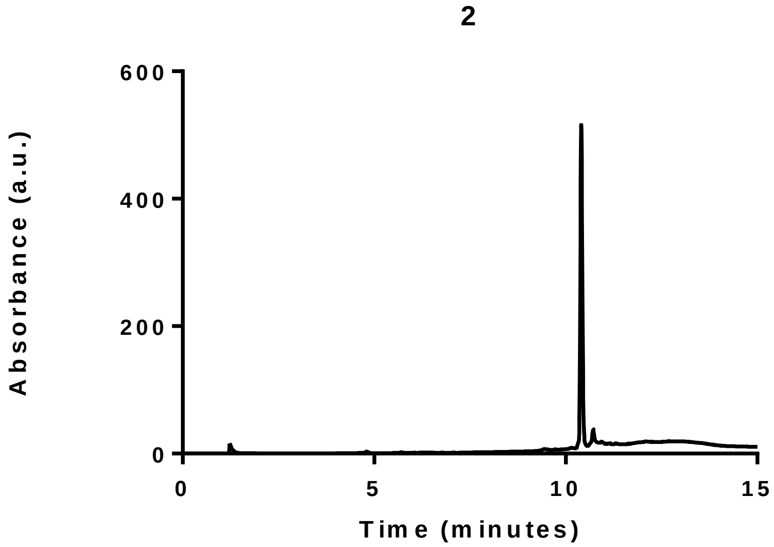

Figure S16. HPLC chromatogram of **2,** (λ = 280 nm).

Electrochemistry:

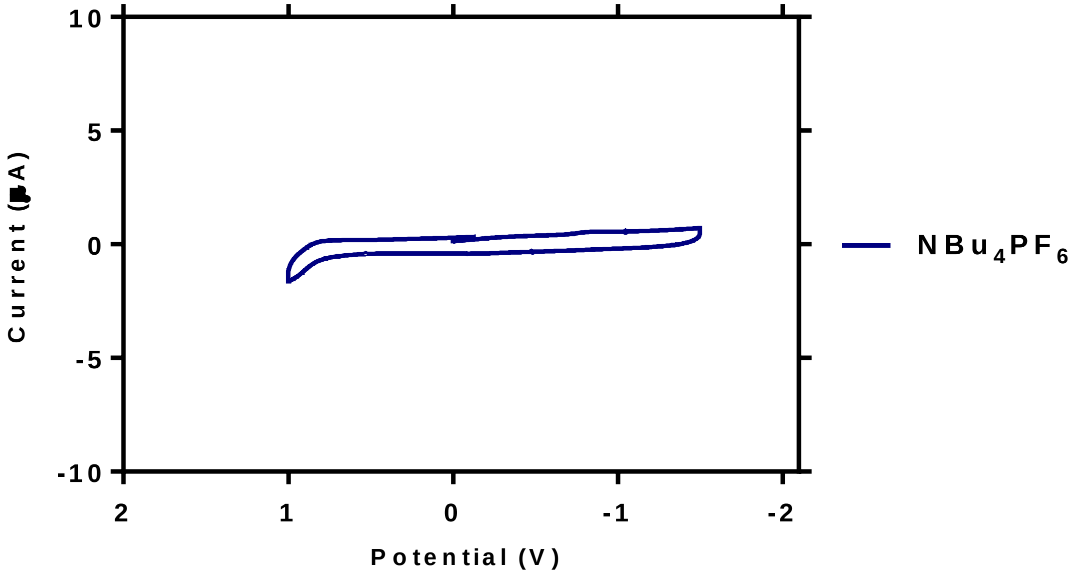

Figure S17. Cyclic voltammogram of the electrolyte (NBu_4PF_6) performed at room temperature at 100 mV/s scan rate. The potential is referenced to Ag/AgCl.

X-ray Accession Code: CCDC 2044732 contain the supplementary crystallographic data for this paper. These data can be obtained free of charge via www.ccdc.cam.ac.uk/data_request/cif, or by emailing data_request@ccdc.cam.ac.uk, or by contacting The Cambridge Crystallographic Data Centre, 12 Union Road, Cambridge CB2 1EZ, UK; fax: +44-1223-336033.
